# Skeletal ^18^F-PSMA-1007 uptake in prostate cancer patients

**DOI:** 10.1177/17588359231179311

**Published:** 2023-06-29

**Authors:** Jorinde Janssen, Walter Noordzij, Ton Velleman, Igle Jan de Jong, Johannes A. Langendijk, J. Fred Verzijlbergen, Gilles N. Stormezand, Shafak Aluwini

**Affiliations:** Department of Radiation Oncology, University of Groningen, University Medical Center Groningen, Hanzeplein 1, Groningen 9713GZ, The Netherlands; Department of Radiology and Nuclear Medicine, University of Groningen, University Medical Center Groningen, Groningen, The Netherlands; Department of Radiology and Nuclear Medicine, University of Groningen, University Medical Center Groningen, Groningen, The Netherlands; Department of Urology, University of Groningen, University Medical Center Groningen, Groningen, The Netherlands; Department of Radiation Oncology, University of Groningen, University Medical Center Groningen, Groningen, The Netherlands; Department of Radiology and Nuclear Medicine, Radboud University Medical Center, Nijmegen, The Netherlands; Department of Radiology and Nuclear Medicine, University of Groningen, University Medical Center Groningen, Groningen, The Netherlands; Department of Radiation Oncology, University of Groningen, University Medical Center Groningen, Groningen, The Netherlands

**Keywords:** ^18^F-PSMA-1007, interobserver agreement, prostate cancer, PSMA PET/CT, skeletal uptake

## Abstract

**Background/objectives:**

Accurate and uniform interpretation and reporting of metastatic prostate cancer (PCa) lesions on prostate-specific membrane antigen (PSMA) positron emission tomography/computed tomography (PET/CT) are indispensable. ^18^F-PSMA-1007 is increasingly used because of its favorable imaging characteristics. However, increased non-specific skeletal uptake may be an important pitfall of this radioligand. Therefore, we aimed to assess the interobserver variation in reporting skeletal ^18^F-PSMA-1007 uptake on PET/CT.

**Design/methods:**

In total, 33 ^18^F-PSMA-1007 PET/CT scans of 21 patients with primary PCa and 12 patients with biochemical recurrence were included, and a total of 85 skeletal lesions were evaluated by three independent observers. The primary endpoint was the interobserver variability of the likelihood of malignancy of the skeletal lesions on both patient and lesion level (kappa analysis).

**Results:**

Observers qualified most lesions as not malignant (81–91%) and the overall mean interobserver agreement was moderate on both patient (κ: 0.54) and lesion level (κ: 0.55). In 52 lesions without corresponding CT substrate, the rating resulted in not malignant in 95–100%. Availability of additional imaging (60% of lesions) did not improve interobserver agreement (κ: 0.39 on lesion level) and resulted in unchanged rating for all observers in 78%.

**Conclusion:**

This interobserver analysis of skeletal ^18^F-PSMA-1007 uptake resulted in moderate agreement, in line with rates reported in literature. Importantly, the presence of non-specific skeletal uptake without CT substrate, as a potential shortcoming of ^18^F-PSMA-1007, did not impair interobserver agreement.

## Introduction

Positron emission tomography (PET) imaging using radiolabeled ligands targeted at the prostate-specific membrane antigen (PSMA) is increasingly utilized as a diagnostic imaging tool for prostate cancer (PCa), and is the imaging modality of choice in primary high-risk and biochemically recurrent PCa.^1,2^ In case of biochemical recurrence, the PSMA PET/computed tomography (CT) contributes to early detection of metastases at a low prostate-specific antigen (PSA) level leading to management decision adjustment in >50% of patients.^3,4^

Advanced personalized PCa treatment modalities such as metastasis-directed radiotherapy and PSMA radioligand therapy are highly dependent on PSMA PET/CT reported findings.^5–7^ Therefore, accurate image interpretation plus complete and uniform reporting of findings on PSMA PET/CT are mandatory and indispensable. The ^68^Ga-labeled PSMA agent is the worldwide most commonly used PSMA radiotracer in trials and in clinic practice, and multiple studies reported high sensitivity and specificity for ^68^Ga-PSMA.^8^ Nevertheless, many centers are replacing the ^68^Ga-PSMA radiotracer with ^18^F-labeled PSMA because of several advantages such as longer half-life, improved image resolution, lower urinary clearance, and lower costs.^9^ The standard application of PSMA PET/CT in PCa management policy and the substantial effect on treatment choice in combination with the increasing use of ^18^F-PSMA-1007 require more knowledge of ^18^F-PSMA-1007 uptake patterns and interpretations.

The number of lesions with a (possible) benign origin was reported to be higher in ^18^F-PSMA-1007 compared to ^68^Ga-PSMA.^10^ In some cases, a benign substrate such as an old fracture or degeneration can be recognized on the low-dose CT scan. ^18^F-PSMA-1007 skeletal uptake without substrate on low-dose CT (non-specific) could lead to an increase in disagreement between observers and, subsequently, could make clinical decision more complicated.^10,11^ The lack of data focusing specifically on this subject highlights the urgent need to investigate the impact of skeletal (non-specific) ^18^F-PSMA-1007 uptake in PCa patients. This study describes the interobserver variation of three experienced observers in the assessment of skeletal ^18^F-PSMA-1007 uptake on PET/CT with the aim to assess pitfalls in using ^18^F-PSMA as basis for PSMA-guided therapy.

## Methods

### Patient population

In total, 33 PCa patients (21 with primary diagnosis and 12 with biochemical recurrence) with at least one focus of skeletal ^18^F-PSMA-1007 tracer uptake were included in this retrospective analysis. Available additional diagnostic imaging (MRI, bone scan, CT scan) was identified from the electronic patient file and collected.

### PSMA PET/CT imaging

The ^18^F-PSMA-1007 PET/CT imaging was performed between April 2019 and January 2021 at three nuclear medicine departments in the Netherlands (20%, 32%, and 48% of patients, respectively). The ^18^F-PSMA-1007 radioligand was synthesized following the procedure described by Cardinale *et al.*^12,^18^^ F-PSMA-1007 was administered as an intravenous bolus injection (mean 198 ± 49 MBq, range 119–350 MBq). PET images were acquired after 60–120 min (±5 min) in 3D mode with an acquisition time of 3–4 min per position. The full information on image correction and reconstruction has been added as Supplemental Table 1. Low-dose CT was acquired from skull vertex to mid-thigh. Emission data were corrected and reconstructed according to the local protocol.

### Imaging analysis

Three independent observers evaluated the 85 skeletal lesions on ^18^F-PSMA-1007 PET/CT scans. These observers were two expert nuclear medicine physicians (O1 and O2, >10 years of nuclear medicine experience, 7 years of experience in reporting PSMA PET/CT) and one expert radiologist (O3, 10 years of musculoskeletal radiology experience and 1 year of experience in reporting PSMA PET/CT). All ^18^F-PSMA-1007 PET/CT scans were anonymized prior to evaluation, and the observers were only provided with information about the anatomical location of each lesion (no clinical patient data).

#### Lesion report

The image analysis was structured in a lesion report according to the European Association of Nuclear Medicine guideline.^13^ The report included a five-point scale, rating five different topics: image quality, level of noise, level of lesion uptake, substrate on low-dose CT, and likelihood of malignancy (Table 1). Lesion reports were completed by each observer in a REDCap™ database and reciprocally blinded for the other observers to avoid bias.

**Table 1.** The structure of the answers required in this study.

Question	Answers				
Part 1 – No additional imaging available (*n* = 85)					
1. Image quality	Excellent	Good	Fair	Poor	Very poor
2. Level of noise	Excellent	Good	Fair	Poor	Very poor
3. Level of lesion uptake	Excellent	Good	Fair	Poor	Very poor
4a. Presence of substrate on CT	Yes	No			
4b. If 4a answers yes: Likelihood of malignancy of substrate on CT	Definitely	Probably	Equivocal	Probably not	Definitely not
5. Likelihood of malignancy	Definitely	Probably	Equivocal	Probably not	Definitely not
Part 2 – With additional imaging available (*n* = 51)					
1. Likelihood of malignancy (combining all available imaging)	Definitely	Equivocal	Definitely not		

After completion of part 1, the database was locked and data reports were blinded for all observers. The role of additional imaging was evaluated by a separate question considering likelihood of malignancy in a three-point scale rating. A total of 51 lesions with available additional imaging from the patient record were re-analyzed to support the final finding.

### Statistics

The interobserver variation was reported on patient (lowest score per patient) and lesion level using an index of observer agreement, kappa analysis (unweighted).^14^ For the kappa analysis, the five-point scale in the first report was clustered into three groups: Malignant (1, 2: Definitely and Probably) | Equivocal | Not malignant (4, 5: Probably not and Definitely not). The three-point scale in the second report was converted similarly: Malignant (Definitely), Equivocal, Not malignant (Definitely not). The kappa was interpreted as fair (>0.20), moderate (>0.40), substantial (>0.60), and almost perfect agreement (>0.80).^14^

## Results

**Figure 1.**
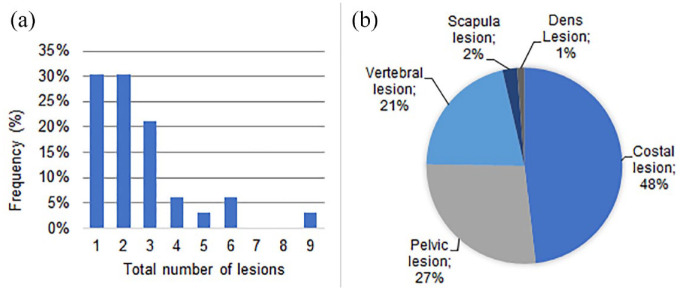
Lesion number and lesion location. (a) Frequency diagram representing the number of lesions per patient (*n* = 33). (b) Pie chart representing the distribution of lesion locations (*n* = 85).

In total, 85 ^18^F-PSMA-1007 avid skeletal lesions in 33 patients were identified from the original nuclear medicine physicians report. The median number of lesions per patient was 2 (range 1–9). Lesions (*n* = 85) were located in ribs (48%), pelvis (27%), vertebra (27%), scapula (2%), and dens (1%; Figure 1(a) and (b)).

For patients with primary PCa (64%), the median PSA value at the time of PSMA PET imaging was 22.0 µg/L (range: 4.7–73.0 µg/L). The median PSA in patients with biochemical recurrence (36%) at the time of PSMA PET was 0.9 µg/L (range: 0.2–5.7 µg/L). A summary of patient demographics is found in Table 2.

**Table 2.** The characteristics of the included 33 patients with primary or recurrent prostate cancer.

Variable	Primary PCa (*n* = 21)	Recurrent PCa (*n* = 12)
PSA (µg/L)	22.0 (4.7–73.0)	0.9 (0.2–5.7)
Primary Gleason score (%)		
⩽7	11/21 (52)	11/12 (92)
8	5/21 (24)	0 (0)
>9	5/21 (24)	1/12 (8)
Primary *T*-stage (%)		
T1–2	14/21 (67)	5/12 (42)
T3	7/21 (33)	7/12 (58)
Primary *N*-stage (%)		
Unknown	0 (0)	1/12 (8)
Nx	10/21 (48)	5/12 (42)
N0	3/21 (14)	3/12 (25)
N1	8/21 (38)	3/12 (25)

PCa, prostate cancer.

### Likelihood of malignancy

Observers qualified most lesions as not malignant (81–91%), 7–15% as malignant, and 1–4% as equivocal. The mean interobserver agreement of overall likelihood of malignancy was 89% on lesion level (mean κ 0.547), and 82% on patient level (mean κ 0.544) (Table 3).

**Table 3.** The agreed number and interobserver agreement on lesion and patient level.

Variable		Agreed number (%)	Kappa statistics (SE)*
Likelihood of malignancy			
Lesion level	O1 *versus* O2	77 (91)	0.536 (0.135)
	O2 *versus* O3	74 (87)	0.489 (0.121)
	O1 *versus* O3	76 (89)	0.615 (0.115)
	Mean	76 (89)	0.547
Likelihood of malignancy			
Patient level	O1 *versus* O2	29 (88)	0.634 (0.151)
	O2 *versus* O3	27 (82)	0.422 (0.146)
	O1 *versus* O3	25 (76)	0.575 (0.149)
	Mean	27 (82)	0.544

* The unweighted kappa was applied to three categories (not malignant, equivocal, and malignant) in all likelihood of malignancy analyses. The unweighted kappa assigns an equal value to all categories.

### Image quality, level of noise, and uptake

**Figure 2.**
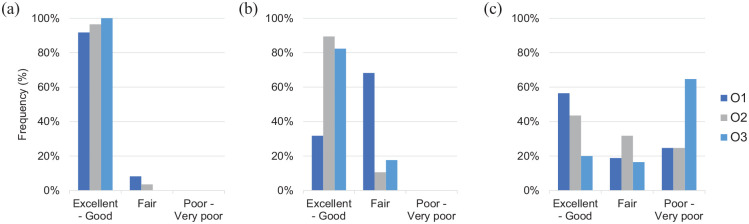
Representation of frequencies of rating of image quality, level of noise, and level of uptake in report 1 by three observers. O1, Observer 1; O2, observer 2; O3, Observer 3.

Image quality of the PSMA PET/CT was rated good to excellent in most cases (92–100%), and level of noise was rated fair to good in 89–100% of cases. Meanwhile, lesion uptake showed more broad variation between observers ranging from mostly excellent/good to mostly poor/very poor (Figure 2).

Lesions with excellent to good uptake were rated malignant in 27% (8/30, median of 3 ratings), and lesions with poor to very poor uptake were never rated malignant (0/27, median of 3 ratings).

### Corresponding CT substrate

Substrate on low-dose CT was rated as present by at least two observers in 21 lesions (25%), and these lesions with substrate were rated malignant in 43% (9/21), and not malignant in 52% (agreement of 71%, *k* = 0.489). Substrate on corresponding CT was absent in 64 lesions (75%), and their rating was not malignant in 100%, 98%, and 94% of lesions per observer, respectively (agreement 95%).

**Figure 3.**
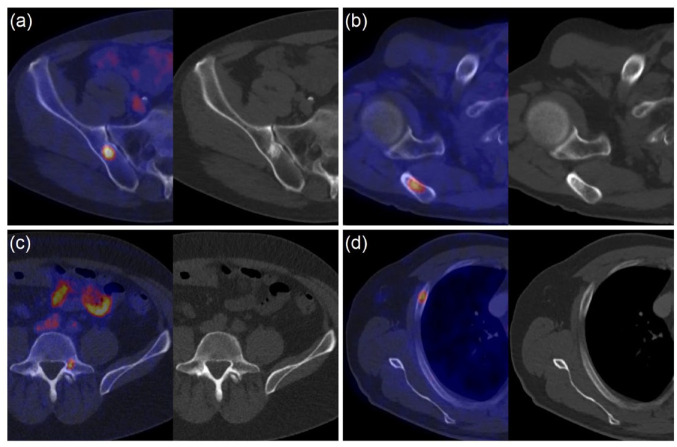
Four examples of lesions on fused PSMA PET/CT (left) and low-dose CT (right): (a) iliac lesion with corresponding CT substrate rated as malignant, (b) scapula lesion with corresponding CT substrate rated as malignant, (c) vertebral lesion with corresponding CT substrate rated as not malignant (substrate corresponding to vertebral hemangioma), and (d) costal lesion without corresponding CT substrate rated as not malignant. PET/CT, positron emission tomography/computed tomography; PSMA, prostate-specific membrane antigen.

Four examples of lesions with and without corresponding CT substrate are demonstrated in Figure 3.

### Additional imaging

Additional imaging was available for 51 lesions in 28 patients; this included 36 lesions on diagnostic CT (71%), 19 previous PET/CT scans (37%), 10 MRI scans (20%), and 4 bone scans (8%). For 17 out of 51 lesions (33%), more than one additional image set was available.

Lesions with additional imaging were rated not malignant in 78–88% and interobserver agreement was 80% (mean κ 0.385), which is similar to the rating for these same lesions without the use of additional imaging (report part 1) (77–92% not malignant, agreement 84%; Table 4).

**Table 4.** Results for lesions with additional imaging present (*n* = 51): the agreed number and interobserver agreement.

Report		Agreed number (%)	Kappa statistics (SE)*
Report 1: Additional imaging present, not available for observers			
Likelihood of malignancy	O1 *versus* O2	88	0.405 (0.174)
	O2 *versus* O3	82	0.378 (0.140)
	O1 *versus* O3	82	0.446 (0.152)
	Mean	84	0.410
Report 2: Additional imaging present AND available for observers			
Likelihood of malignancy	O1 *versus* O2	80	0.335 (0.123)
	O2 *versus* O3	84	0.350 (0.118)
	O1 *versus* O3	76	0.471 (0.146)
	Mean	80	0.385

* The unweighted kappa was applied to three categories (not malignant, equivocal, and malignant); the unweighted kappa assigns an equal value to all categories.

Furthermore, the availability of additional imaging resulted in identical likelihood of malignancy in 78% of lesions (40 out of 51) by all observers (report 1 compared to report 2).

## Discussion

This analysis demonstrated moderate interobserver agreement in malignancy rating of skeletal ^18^F-PSMA-1007 uptake on lesion and patient level (agreement 89% and 82%, respectively).

Lesions with poor uptake were never rated malignant (100%). Lesions without CT substrate were rated ‘not malignant’ in 97% and interobserver agreement was 95%. Therefore, non-specific skeletal uptake, often mentioned as a potential shortcoming of ^18^F-PSMA-1007, did not hamper interobserver agreement.

Interobserver agreement results in this report are in line with reported analysis for ^18^F-PSMA-1007 (74–94% agreement for bone lesions) and with reported agreement for ^68^Ga-PSMA (κ: 0.559 for bone lesions).^15,16^

Data reporting interobserver agreement for skeletal PSMA uptake regularly describe agreement per patient or per region and not per lesion.^15–18^ Our analysis showed a moderate interobserver agreement of skeletal ^18^F-PSMA-1007 uptake on lesion level (mean agreement 89%, mean κ: 0.55). A uniform rating on lesion level is crucial since clinical decision-making is mostly based on a lesion level, especially in tailored therapy such as metastasis-directed radiotherapy and PSMA radioligand therapy.^2,5,7^

Skeletal PSMA uptake with corresponding CT substrate is not always associated with malignancy, but has also been observed in benign disease such as fracture, degeneration, or hemangioma.^13,19,20^ In this report, lesions with corresponding substrate were rated benign in 52% and in 30% of lesions with substrate the observers disagreed on the nature of lesions. This variation in differentiation between malignant and benign in ^18^F-PSMA-1007 uptake with CT substrate reflects the difficulty to give a conclusive report on the nature of the lesion in the absence of pathological validation and highlights the need for more guidance on ^18^F-PSMA-1007-specific reporting. Further research supported by hard evidence such as pathological confirmation (biopsy) and changes on follow-up scans is urgently needed.

Access to other imaging techniques and detailed clinical patient information reported to positively contribute to a more conform interobserver agreement.^21^ Nevertheless, we reported no improvement in interobserver agreement if additional imaging was available (in 78% of lesions with accessible conventional imaging no change in rating was reported). However, in our report, observers were blinded from any clinical patient data to ensure unbiased interpretation. Apparently, the availability of both extra imaging and clinical patient information is important to improve interobserver agreement. Therefore, our reported interobserver agreement might be an underestimation of daily practice when all additional imaging and patient data are available, which also emphasizes the importance of providing nuclear medicine physicians with accurate clinical information.

Published interobserver analyses are regularly based on a two-point scale (malignant *versus* not malignant).^16,18,22^ However, the three PSMA PET/CT interpretation guidelines currently used in clinical practice (E-PSMA, PSMA-RADS, and PROMISE) strongly recommend the use of a three-point scale: Malignant, Equivocal, and Not malignant.^13,23,24^ Therefore, the use of a three-point scale based rating of skeletal lesions in this analysis is representative and informative for daily practice.

Limitations of this study include the retrospective nature of the data, the lack of pathological confirmation (small lesions, 75% without substrate and, therefore, prone to sample error), and the relatively low number of patients. This research contributed in gaining more insight in ^18^F-PSMA-1007 skeletal uptake interpretations and possible pitfalls. Nevertheless, more research is necessary, preferably in a prospective or randomized context.

## Conclusion

This analysis of interobserver variation in reporting skeletal ^18^F-PSMA-1007 uptake resulted in a moderate, clinically acceptable interobserver agreement in line with rates reported in literature. Importantly, the presence of non-specific ^18^F-PSMA-1007 skeletal uptake without CT substrate, a dreaded pitfall of ^18^F-PSMA-1007, did not hamper interobserver agreement.

## Supplemental Material

sj-docx-1-tam-10.1177_17588359231179311 – Supplemental material for Skeletal 18F-PSMA-1007 uptake in prostate cancer patients Click here for additional data file. Supplemental material, sj-docx-1-tam-10.1177_17588359231179311 for Skeletal 18F-PSMA-1007 uptake in prostate cancer patients by Jorinde Janssen, Walter Noordzij, Ton Velleman, Igle Jan de Jong, Johannes A. Langendijk, J. Fred Verzijlbergen, Gilles N. Stormezand and Shafak Aluwini in Therapeutic Advances in Medical Oncology

## Declarations

**Ethics approval and consent to participate:** The Medical Ethics Review Board of the University Medical Center Groningen (METc UMCG) has reviewed the research proposal and concluded that this research does not fall within the scope of the Medical Research Involving Human Subjects Act (WMO). Therefore, the requirement for informed consent to participate has been waived by the METC UMCG. Patients included in this analysis have provided department-specific written informed consent for retrospective data research and/or did not apply for the institutional opt-out registration. An exemption letter of the Medical Ethics Review Board was obtained (METc 2021/470, 08/11/2021).

**Consent for publication:** Not applicable.

**Availability of data and materials:** The datasets generated during and/or analyzed during the current study are available from the corresponding author on reasonable request.
