# Supplementary material for: Skeletal 18F-PSMA-1007 uptake in prostate cancer patients
Source: Ther Adv Med Oncol. 2023 Jun 29;15:17588359231179311. doi: 10.1177/17588359231179311 (PMC10333984; doi:10.1177/17588359231179311)
Supplement: sj-docx-1-tam-10.1177_17588359231179311 – Supplemental material for Skeletal 18F-PSMA-1007 uptake in prostate cancer patients [file sj-docx-1-tam-10.1177_17588359231179311.docx]

Supplementary table 1. PSMA PET/CT Image correction and reconstruction

| Number of patients | Manufacturer Model name | Convolution Kernel | Reconstruction method | Slice Thickness | Rows x collumns | Image delay  (time post injection) |
| --- | --- | --- | --- | --- | --- | --- |
| 2 | Biograph mCT 20 | 5 mm Gaussian filter | 2 iterations 21 subsets | 3.0 mm | matrix 200x200 | 120 min |
| 4 | Biograph mCT 20 | 5 mm Gaussian filter | 3 iterations 21 subsets | 3.0 mm | matrix 200x200 | 120 min |
| 2 | Biograph Vision | 5 mm Gaussian filter | 4 iterations 5 subsets | 2.0 mm | matrix 220x220 | 60 min |
| 2 | Biograph Vision | 7 mm Gaussian filter | 4 iterations 5 subsets | 2.0 mm | matrix 440x440 | 60 min |
| 2 | Biograph Vision | 5 mm Gaussian filter | 3 iterations 5 subsets | 2.0 mm | matrix 440x440 | 60 min |
| 1 | Biograph mCT 64 | 6.5 mm Gaussian filter | 3 iterations 21 subsets | 2.0 mm | matrix 256x256 | 60 min |
| 1 | Biograph mCT 64 | 5 mm Gaussian filter | 3 iterations 24 subsets | 2.0 mm | matrix 200x200 | 60 min |
| 19 | Biograph mCT 64 | 5 mm Gaussian filter | 3 iterations 21 subsets | 2.0 mm | matrix 400x400 | 60 min |
